# Local adaptation to temperature and precipitation in naturally fragmented populations of *Cephalotaxus oliveri*, an endangered conifer endemic to China

**DOI:** 10.1038/srep25031

**Published:** 2016-04-26

**Authors:** Ting Wang, Zhen Wang, Fan Xia, Yingjuan Su

**Affiliations:** 1 College of Life Sciences, South China Agricultural University, Guangzhou 510642, China; 2 College of Sciences, Nanjing Agricultural University, Nanjing 210095, China; 3 School of Life Sciences, Sun Yat-sen University, Guangzhou 510275, China

## Abstract

*Cephalotaxus oliveri* is an endangered tertiary relict conifer endemic to China. The species survives in a wide range from west to east with heterogeneous climatic conditions. Precipitation and temperature are main restrictive factors for distribution of *C. oliveri*. In order to comprehend the mechanism of adaptive evolution to climate variation, we employed ISSR markers to detect adaptive evolution loci, to identify the association between variation in temperature and precipitation and adaptive loci, and to investigate the genetic structure for 22 *C. oliveri* natural populations. In total, 14 outlier loci were identified, of which five were associated with temperature and precipitation. Among outlier loci, linkage disequilibrium (LD) was high (42.86%), which also provided strong evidence for selection. In addition, *C. oliveri* possessed high genetic variation (93.31%) and population differentiation, which may provide raw material to evolution and accelerate local adaptation, respectively. Ecological niche modeling showed that global warming will cause a shift for populations of *C. oliveri* from south to north with a shrinkage of southern areas. Our results contribute to understand the potential response of conifers to climatic changes, and provide new insights for conifer resource management and conservation strategies.

Global climates including temperatures and precipitation are rapidly changing. The changes of temperature mainly embody in variations in the daily, regional, seasonal, and annual mean temperatures as well as the increase of the intensity, frequency, and duration of abnormally low and high temperatures^1^. The alterations in precipitation primarily reflect in seasonal and temporal variability with the number of precipitation days and the length of dry spells^2^. It is noted that rapid changes in temperature and precipitation exert new selection pressures on plant populations^3^, which strongly influences their physiology, abundance, and distribution^4^. For instance, when plants encounter temperature extremes and extreme events including droughts and storms, flowering which is highly relevant for plant genetic adaptation to climate change is significantly ahead of time^5^. In this context, changes in temperature and precipitation provide an opportunity to investigate plant evolutionary adaptation.

Temperature and precipitation have been revealed as important drivers for local adaptation for conifers^6^. Temperature can profoundly influence seed germination, seedling growth, productivity and distribution of conifers, whereas precipitation has a determinative impact on variables such as soil moisture and the length of a wet or dry season^7^,^8^. Temporal and spatial variation in temperature and precipitation can also strongly influence the survival of conifers^9^. In extreme situations, high or low temperature stresses and precipitation events will ultimately cause mortality. Thus how to cope with temperature and precipitation becomes particularly important for conifers^10^. Due to their immobility and limited gene flow among populations, conifers locally have to adapt to fluctuations of temperature and precipitation by change of genotypes^11^,^12^ and inevitably leaves adaptive imprints at the genomic level. Indeed adaptive fingerprints have been widely detected in conifers. For instance, six SNPs in five climate-related candidate genes have been shown under divergent selection between two closely related species *Pinus massoniana* and *Pinus hwangshanensis*^6^. Twenty-three candidate SNPs related to temperature and precipitation have been identified in black spruce (*Picea mariana*)^13^. The prime candidate genes for adaptation to climatic variation involves in a variety of putative functions, including phenology, growth, reproduction, wood formation, lignin metabolism, and stress response^4^. Moreover, conifers also possess specific characteristics such as low domestication, large open-pollinated native populations, and high levels of genetic variation^14^, which makes them particularly valuable for examining local adaptation evoked by temperature and precipitation^14^. Investigating the adaptability of conifers to the two factors may also help to predict their response to future climate change^15^.

*Cephalotaxus oliveri* is an endangered tertiary relict conifer endemic to China with important economical and medical values^16^,^17^. The plant is ascribed to the Genus *Cephalotaxus* sect. *Pectinate* in Cephalotaxaceae^17^. *Cephalotaxus oliveri* is a wind-pollinated dioecious woody shrubs or small trees up to 4 m tall with yellow to grayish brown and scaly bark. Its distinguishing features embody in leaves densely arranged on leafy shoots and stomatal bands on abaxial surface^17^. Due to deforestation and overexploitation, it has been regarded as a vulnerable species by IUCN^17^. This species is essentially undomesticated and has large geographic ranges including montane regions of northern Guangdong, Guizhou, western Hubei, Hunan, eastern Jiangxi, southern and western Sichuan, and eastern Yunnan in China. Its natural populations have long been disjunctly distributed in subtropical evergreen and deciduous broad-leaved forests, where they occupy humid, shady niches at elevations of 300–1800 m with significant climate heterogeneity^17^. Precipitation and temperature are restrictive factors for the distribution of *C. oliveri*^18^. For instance, precipitation, extremely highest temperature, annual average precipitation, monthly highest average temperature, and annual average temperature have been documented to limit the horizontal and vertical distribution of *Cephalotaxus* in Yunnan^19^. Moreover, temperature can also influence the seed and seedling physiology and morphology^20^,^21^ as well as the speed of seed germination in *Cephalotaxus*^22^. *Cephalotaxus oliveri* was hypothesized to originate in the Oligocene and its population diversification was associated with the rapid uplift of the Qinghai-Tibetan Plateau^23^. The plant has experienced severe changes of temperature and precipitation over the past millions of years and well adapts to cold and arid environment. Hence, *C. oliveri* is suitable for investigating the adaptive evolution mechanism to climate variation. More importantly, in the light of global climate change, it is conducive to understanding the adaptive potential of *C. oliveri* and formulating protection strategy.

Due to the lack of genomic resources for nonmodel species, molecular marker-based genomic scans have been widely used to explore adaptive loci. All loci across the genome are generally expected to share the same demographic history^13^. However, when selective pressures result in strong differentiation of allele frequencies at some loci in the genome^24^, these loci will deviate from the equilibrium model and are considered to be potentially adaptive^25^. Currently, two approaches have been frequently applied to detect adaptive loci. One is Bayescan, which uses Bayesian estimation of the coancestry coefficient *F*_ST_ to decide whether a particular locus is adaptive^26^,^27^. The other is Dfdist, which is mainly a frequentist method based on summary statistics of a symmetrical island model to identify the loci under selection^26^,^28^. If adaptive loci are further combined with climatic variables, it may be possible to estimate which climatic factors are responsible for adaptive evolution. The association between allele frequency variation and environmental variables can be evaluated by the Spatial Analysis Method (SAM) through logistic regressions^29^,^30^. The advantages of SAM lie in that it does not depend on genetic models and works at the individual level^29^.

In this study, we employed ISSR markers to detect adaptive loci under selection and utilize SAM to identify the association between climatic variations and genetic data. In addition, we also investigated the genetic structure among natural populations of *C. oliveri.* Because populations sampled represented over a local scale, our results may precisely detect the genetic signatures for adaptation. The goals of the study were (1) to examine adaptive loci in the genome of *C. oliveri*; (2) to analyze the correlation between climatic variables and adaptive loci; (3) to evaluate the population genetic structure of *C. oliveri*, and (4) to explore the genetic basis of the adaptation of *C. oliveri* to climate.

## Results

### Genetic Structure

Twenty-one ISSR primers were selected to investigate the genetic structure in *C. oliveri* populations. Overall, 310 reliable loci were identified with 100% polymorphic ranging in size from 200 to 2000 bp. A high level of genetic variation was observed with 93.31% polymorphic loci at the species level (Table 1). The highest number of polymorphic loci (*PPB* = 78.8%, Hs = 0.2769, I = 0.4125) was exhibited in HNym population and the lowest (*PPB* = 18.44%, Hs = 0.0798, I = 0.1141) in JXxs population. At the regional scale, Hunan (*PPB* = 87.88%, Hs = 0.2435, I = 0.377) maintained the highest level of variation and Guangdong (*PPB* = 44.67%, Hs = 0.1792, I = 0.2607) the lowest. The results indicated that *C. oliveri* possesses a high level of genetic variation.

Populations were significantly structured as revealed by overall *F*_ST_ (0.39565) and *G*_*ST*_ (0.3862). AMOVA results further showed that most genetic variation was occurred within populations (60.44%, *F*_ST_ = 0.39565, *P* < 0.001), whereas the proportion of genetic variation among populations within regions was 29.87% (*F*_SC_=0.33076, *P* < 0.001) (Table 2). Only 9.7% genetic variation occurred among regions (*F*_CT_=0.09696, *P* < 0.001). In addition, significant patterns of isolation by distance were revealed by comparing *F*_ST_ values with geographical distances (r = 0.571069, *P* < 0.001).

**Figure 1:**
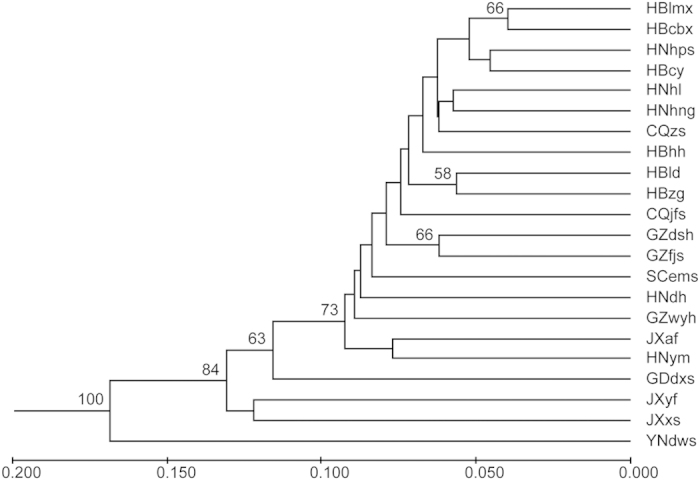
UPGMA dendrogram among 22 populations of *C. oliveri* was constructed based on Nei’s unbiased genetic distances. Bootstrap values larger than 50% were displayed above branches (% of 1000 replicates). Scale between branch lengths and genetic distances was shown at the bottom of figure.

Dendrogram was constructed by the UPGMA method. The results showed that 22 populations of *C. oliveri* were clustered into two groups (Fig. 1). YNdws, the most geographically distant population, was clustered into the separated group. Other 21 populations were clustered into the other group.

**Figure 2:**
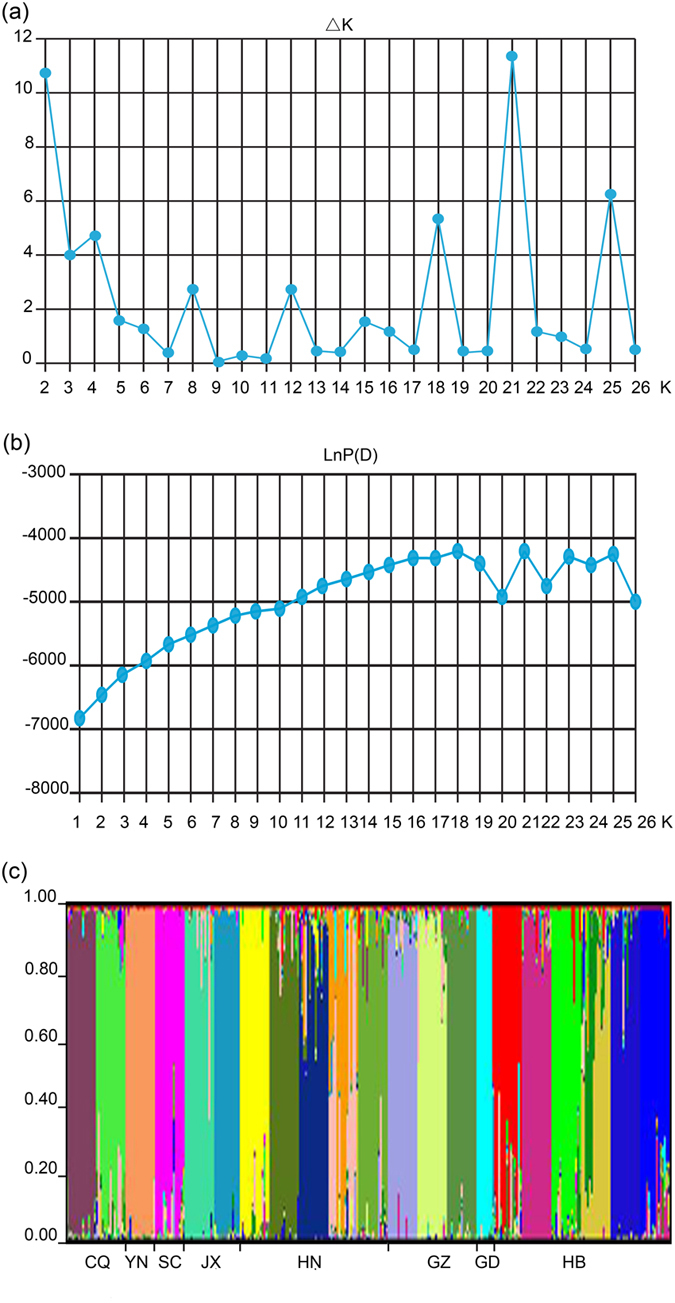
Bayesian structure analyses of *C. oliveri* populations based on ISSR loci. (**a**) Estimates of an ad hoc quantity Δ*K* with respect to *K*. The result for *K *= 21 supported by a high Δ*K* value was presented. (**b**) A plot of the posterior probability of the data (LnP(D)) values for a given *K* (1–26). The 21 represented the value of *K* with the highest likelihood. (**c**) Genetic structure of *C. oliveri* in eight regions for *K *= 21 clusters.

Δ*K* clearly demonstrated that the uppermost *K* equaled 21 (Fig. 2a). The most likely number of genetic clusters *K* from STRUCTURE was also 21 (mean LnP(D) = −39912.5, Var[LnP(D)] = 4877) (Fig. 2b). Populations HBzg and HBld, GZdsh, GZfjs, JXyf, and JXxs were clearly evident, with high proportions of individual assignment to the correct region (Fig. 2c). Only a few populations appeared admixed with others. The results suggested that a high differentiation level existed in *C.oliveri* populations.

### Outlier detection

**Figure 3:**
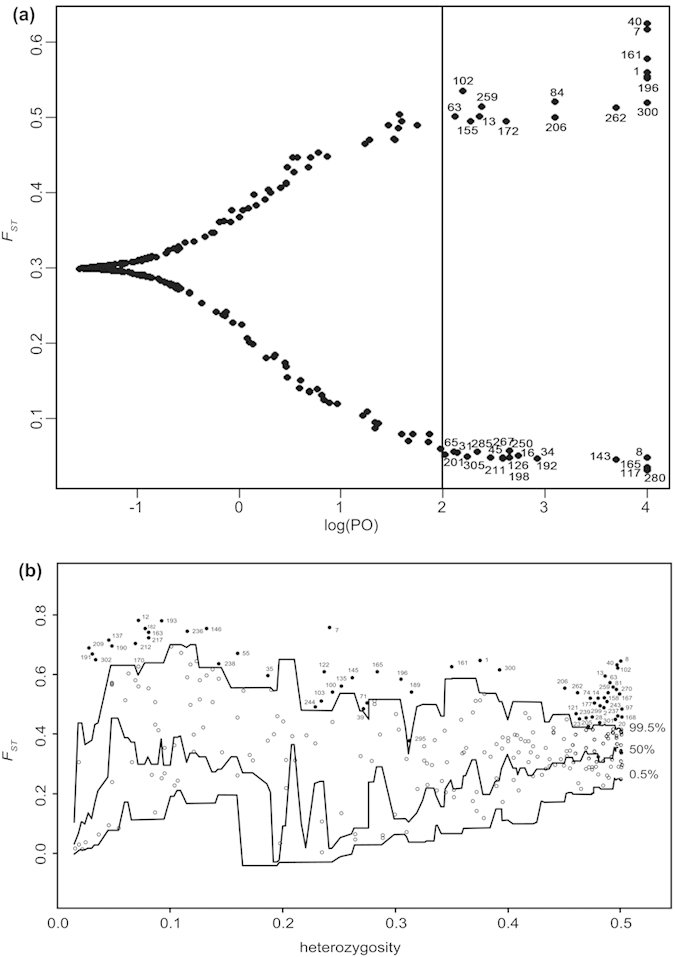
Outlier loci identified by Bayescan and Dfdist. (**a**) Plot of *F*_ST_ values and log_10_PO for 310 loci identified using Bayescan. Lines log_10_PO = 2 indicate “decisive” evidence for selection corresponding to a posterior probability of 0.99. Solid black dots greater than log_10_PO 2 represented outlier loci. (**b**) Outlier detection performed with Dfdist. Plot of *F*_ST_ values of 310 loci in *C. oliveri* populations was against heterozygosity. The 0.5%, 50%, and 99.5% represented confidence intervals, respectively. Loci above the 99.5% line were designated as outlier loci.

**Figure 4:**
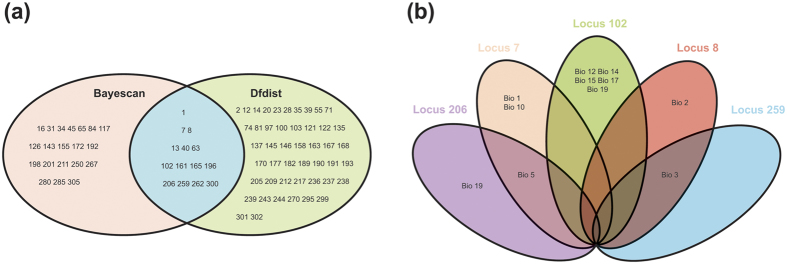
Number summary of outlier loci and significant association between outliers and climatic variables. (**a**) Thirty-two, 61, and 14 outlier loci were detected to be subject selection in *C. oliveri* using Bayescan, Dfdist, and both with Dfdist and Bayescan, respectively. (**b**) The number association between outliers and climatic variables in SAM analysis. Locus 7 and 8 were significantly associated with three and two temperature variables, respectively. Locus 206 was simultaneously linked to two variables of temperature and precipitation. Locus 102 exhibited 5 significant allele–climate variables. Locus 259 was only linked to one climatic factor.

Bayescan identified 32 loci as outliers with a log_10_PO above 2, which is a threshold for decisive evidence for accepting a model under selection, corresponding to a posterior probability greater than 0.99^31^ (Fig. 3a). Using the Dfdist, we detected 61 adaptive loci at the 99.5% confidence level (Fig. 3b). Based on two complementary analyses, 14 outlier loci (1, 7, 8, 13, 40, 63, 102, 161, 165, 196, 206, 259, 262, and 300) were identified, which represented truly adaptive loci (Fig. 4a). The very stringent significance criteria in the two approaches also ensured the robustness of 14 outlier loci.

### Linkage disequilibrium

Linkage disequilibrium (LD) was detected for 28 of the 14225 combinations of all 310 loci with the false discovery rate of 0.1. Twenty of the 310 loci (6.45%) were involved in the detected combinations in LD. In contrast, six (42.86%) of the 14 outlier loci were involved in LD; they were Locus 40, 63, 102, 161, 165, and 206. When two or more linked loci were in LD within a chromosomal region, this region was defined as an LD block^32^. We found that Locus 165 formed LD blocks with nine loci (Locus 130, 135, 144, 149, 150, 151, 152, 154, and 156) (Table 3).

### Association with climatic variables

The logistic regressions of 310 ISSR markers and 19 climatic variables were calculated using the SAM program. With 99.9999% confidence level, on a total of 6510 models computed, SAM identified 20 significant associations (0.31%). Of those, ten loci were significantly related with temperature, and 11 were significantly related to precipitation. Furthermore, five of the 20 loci related with climatic factors were also outlier loci detected by Dfdist and Bayescan (Fig. 4b). Locus 7 was significantly associated with annual mean temperature (Bio 1), max temperature of warmest month (Bio 5), and mean temperature of warmest quarter (Bio 10). Locus 8 showed significant association with mean diurnal range (Bio 2) and isothermality (Bio 3). Locus 102 exhibited 5 significant allele–climate variable associations including annual precipitation (Bio 12), precipitation of driest month (Bio 14), precipitation seasonality (Bio 15), precipitation of driest quarter (Bio 17), and precipitation of coldest quarter (Bio 19). Locus 206 was significantly associated with max temperature of warmest month (Bio 5) and precipitation of coldest quarter (Bio 19). Locus 259 was linked to isothermality (Bio 3). Loci 7 and 206 were linked to max temperature of warmest month (Bio 5); Loci 8 and 259 were associated with isothermality (Bio 3); Loci 102 and 206 were correlated to precipitation of coldest quarter (Bio 19). Locus 206 was simultaneously linked to temperature and precipitation.

### Potential current and future geographic distribution

**Figure 5:**
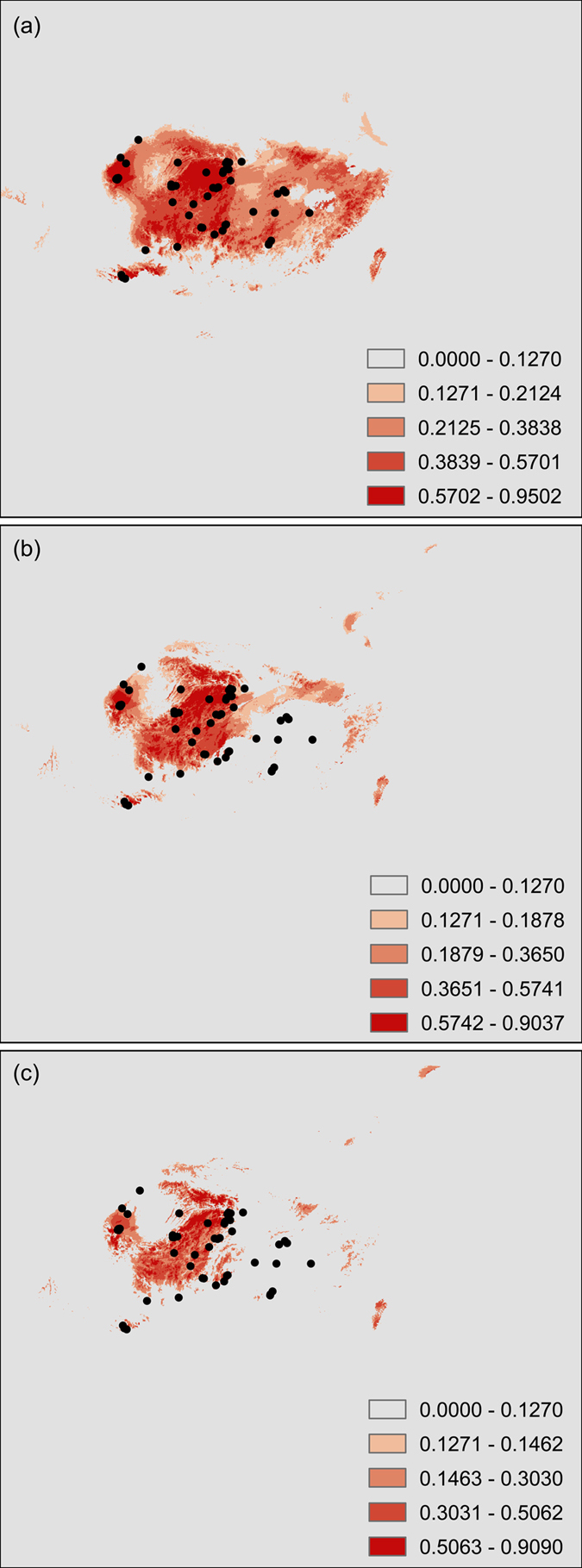
Potential current and future distributions inferred from ecological niche modeling (Program MAXENT 3.3.3k, URL http://www.cs.princeton.edu/~schapire/maxent/.) for *C. oliveri* in China based on WorldClim variables. Two climate scenarios IPCC-CMIP5 RCP 4.5 and RCP 8.5 under HadGEM2-ES model were used to ensure the accuracy of evaluation on future distribution. (a) Predicted current distribution of *C. oliveri* based on climate grids for the periods 1950–2000. (b) Possible future distribution of *C. oliveri* in 2070 based on RCP 4.5 climate scenario. (c) Projected future distribution of *C. oliveri* in 2070 based on RCP 8.5 climate scenario.

Present and future ecological niche models for *C. oliveri* were estimated by MAXENT (Maximum Entropy Model). The average AUC test for replicate runs and the standard deviation were 0.955 and 0.019, respectively, which indicated good predictive model performance. Minimum training presence logistic threshold was 0.1270. The predicted current geographical distribution of *C. oliveri* was generally similar to its actual distribution including Jiangxi, Hunan, Guizhou, Guangxi, and Hubei, even though some predicted areas do not have any records at present (Fig. 5a). These resulting potential distributions are climatically suitable for *C. oliveri*.

The predicted future and current distribution of *C. oliveri* was considerably different in range. The main difference was that the predicted future suitable area showed a significant reduction in comparison with the current one with a general northward range shift (Fig. 5b,c); the present southern and southeastern regions such as Jiangxi, Hunan, Hubei, and Guangxi were predicted to become significantly unfavorable. Loss of suitable habitats indicated a drastically range contraction (Fig. 5b,c). Populations of *C. oliveri* may become more patchily distributed than at present.

## Discussion

This study has analyzed the adaptive evolution of *C. oliveri* to temperature and precipitation through ISSR markers (Inter-Simple Sequence Repeats). ISSRs are highly variable and have been widely applied to the assessment of population genetic diversity and structure in plants^33^,^34^. However, ISSRs were seldom used to detect adaptive loci in genome. Currently, the most efficient approach to identify candidate genomic regions under selection is AFLP (Amplified Fragment Length Polymorphism) based on PCR (Polymerase Chain Reaction) to obtain amplified polymorphisms^35^. Similar to AFLP, ISSR can also generate a large number of polymorphic loci in genome without prior sequence information^33^,^36^,^37^. Although ISSR are assumed to be neutral markers, its primers also can match to microsatellite regions and genes encoding specific proteins^33^,^38^,^39^. Hence ISSR is suitable for detecting candidate loci under selection.

We first applied ISSRs to identify adaptive evolution in *C. oliveri* and ascertain the relationships between candidate loci and climatic factors. In order to ensure unbiased analysis, only distinct, reproducible ISSR loci were scored in this study. Three hundred and ten loci produced by 21 primers have good genome coverage in *C. oliveri*. Fourteen outlier loci were identified by both Dfdist and Bayescan. The two complementary and exhaustive methods guaranteed strong confidence of the 14 loci with very stringent significance criteria. The proportion of outlier loci detected in *C. oliveri* was 4.22%, which conformed to the percentage between 2% to 15% in AFLP genome scan or other molecular markers^40^. For instance, 2.9% in *Mikania micrantha* from AFLP^35^, 3% in Norway spruce (*P. abies*) from RAPD markers^41^, and 3.7% in white spruce from SNPs^42^.

Temperature and precipitation have been identified to be the major selective pressure driving plant adaptation^8^,^9^,^43^,^44^,^45^. The two climatic factors are very important for plant growth, development, survival, reproduction and defense^8^. Currently, adaptive loci associated with temperature and precipitation have been detected in plants^9^. For instance, the close relationship between AFLP allele frequencies and temperature and precipitation have been found in *P. monticola* and *Keteleeria davidiana* var. *formosana*^9^,^43^,^46^. Nine SNPs associated with climate-related complex trait variation have also been identified in Sitka spruce (*P. sitchensis*)^47^. SNPs associated with seasonal minimum temperatures are detected in four conifers, *Abies alba, Larix decidua, P. cembra*, and *P. mugo*, whereas SNPs in *L. decidua* and *P. cembra* are found to be related to seasonal maximum temperature and winter and autumn precipitations^48^. The local polymorphism patterns of candidate genes linked to drought tolerance has also been detected in a widespread Mediterranean conifer (*P. halepensis*)^49^. As restrictive factors, temperature and precipitation were suggested to strongly influence the geographical distribution of *C. oliveri*^18^. In this study, five among 14 outlier loci were also revealed to be associated with temperature or precipitation. Local climatic conditions impacted 1.6% of ISSR loci in *C. oliveri*, suggesting that a relatively small number of loci govern climatic adaptation in this species. The result was very similar to previous studies^13^,^50^. Twenty-two adaptive loci associated with climatic variables was identified in Loblolly pine (*P. taeda*)^50^, whereas ten outlier loci were detected in black spruce (*P. mariana*)^13^. Their proportions of detected loci associated with the climatic variables were 1.3% and 1.7%, respectively.

Furthermore, we found that one adaptive locus simultaneously was linked to temperature and precipitation. The result indicated that the candidate locus might have undergone the same selective pressures^51^. The same phenomenon was also demonstrated in black spruce (*P. mariana*), whose four of 26 outlier SNPs were common to both the temperature and the precipitation^13^. It has been noted that physiological processes involved in adaptation to temperature and precipitation may be related in conifers^52^,^53^. In *P. abies*, drought tolerance was found to be genetically correlated with tolerance to freezing temperature^53^. In *P. mariana*, a gene coding for the DnaJ heat shock protein was detected to carry an adaptive SNP related to both temperature and precipitation, and the protein was produced under stresses involving temperature and moisture^10^. In whitebark pine (*P. albicaulis*) and loblolly pine (*P. taeda*), annual, seasonal mean temperatures, and rainfall patterns also appear to drive local adaptation, which primarily involved response to both temperature and drought^50^,^54^. *Cephalotaxus oliveri* extends from the west to the east in China, with an environmental gradient showing significant precipitation and temperature differences^55^. The finding of adaptive loci implies that *C. oliveri* has successfully responded and adapted to historic climate changes. The species was inferred to originate in the Oligocene [ca. 28.32 million years ago (Ma)] and diversified in the early Miocene (ca. 17.73 Ma)^23^. In the long evolutionary process, the high genetic variation possessed by *C. oliveri* may have provided raw material for its adaptation to changing climatic conditions. Meanwhile, its high population differentiation possibly also accelerated local adaptation. Our results indicated that *C. oliveri* populations well adapt to temperature and precipitation factors including annual mean temperature (Bio 1), mean diurnal range (Bio 2), isothermality (Bio 3), max temperature of warmest month (Bio 5), mean temperature of warmest quarter (Bio 10), annual precipitation (Bio 12), precipitation of driest month (Bio 14), precipitation seasonality (Bio 15), precipitation of driest quarter (Bio 17), and precipitation of coldest quarter (Bio 19) (Fig. 4b).

The geographic pattern also provided a useful indication of adaptive variation^56^,^57^. The natural populations of *C. oliveri* have a wide geographic range, from south to north is from 25°3′N, 130°45′E to 34°40′N, 111°02′E and east to west from 28°37′N, 114°54′E to 27°37′N, 113°52′E (Table 1). Within the range, *Cephalotaxus oliveri* has to face with a great climate variation, including subtropical monsoon climate, East Asian humid monsoon climate, subtropical mountain monsoon humid climate, and eastern humid mountain monsoon climate, respectively^55^, which results in seasonal difference of precipitation. Water availability is one of the major abiotic stressors that can lead to adaptive variation in conifers^50^. Specifically, lack of precipitation during winter period represents a great threat to conifers^58^. In line with this, precipitation of coldest quarter becomes the most often climate variable that was detected in the significant allele-environment associations^57^. In this study, SAM analysis further showed that precipitation of coldest quarter was one of the factors driving the adaptive evolution of *C. oliveri*, which was consistent to the previous ecological hypothesis^55^.

Linkage disequilibrium (LD) in the genomic region can reflect the genetic signature associated with local adaptation, especially for long-lived plants^51^,^59^. Here we observed that of all the 310 loci examined for *C. oliveri*, only twenty (6.45%) were found to be involved in LD. Our result lends further support to the theory that LD in forest trees decays rapidly^60^. However, if only considering outlier loci, the proportion (42.86%) of loci involved in LD was relatively high. This is not unexpected as strong positive selection may increase the frequency of an advantageous allele, causing linked loci remain in strong LD with that allele (genetic hitch-hiking)^59^. More importantly, Locus 165 was identified to form LD blocks with nine loci. The significant LD among the loci reflect that they may not only have experienced the same selection pressure, but also have been acted upon by evolutionary mechanisms like co-adaptation of gene complexes^51^,^61^. The LD blocks implies potential genomic regions that are associated with adaptations.

It is also worthy of note that for the pairwise LD analysis between Locus 102 and 79, 165 and 150, and 206 and 180, the two statistics *D*’ and *r*^2^ acted quite differently; namely, *D*’ had a value of 1, but *r*^2^ were much smaller (Table 3). This performance difference is due to the fact that *D*’ and *r*^2^ reflect different aspects of LD^62^. *D*’ measured only recombinational history, whereas *r*^2^ summarized both recombinational and mutational history. The results of *C. oliveri* indicated that the polymorphisms between the three pairs of locus were not completely correlated, but there was no evidence of recombination.

We used MAXENT to project the distribution of *C. oliveri* under current and future climate conditions. MAXENT captured well a major portion of current distribution of *C. oliveri* in China and also deduced its future range under a climatic warming scenario. With a rate of rising of 0.1 ^o^C–0.4 ^o^C per decade^63^, future temperature was assumed to increase by 2.3 ^o^C–2.7 ^o^C in 2070. As a result, the projection predicted that *C. oliveri* will lose considerable suitable areas with climate warming. Similar predictions have been made for other tree species^54^,^64^,^65^. More specifically, the southern and southeastern populations of *C. oliveri* were projected to be more sensitive to climate warming than others. This information is quite helpful for formulating a protection strategy when considering future climate conditions. Although *C. oliveri* possesses high levels of population genetic variation, its long generation time and limited seed dispersal will constrain adaptations to rapid climate change^54^,^64^,^66^,^67^. On the other hand, at the regional scale Hunan had the highest ISSR variation, whereas the lowest was found in Guangdong. In our previous research, the similar variation pattern has also been revealed for *C. oliveri* populations based on *trn*L-F, *atp*B-*rbc*L and *trn*D-*trn*T sequences^23^. Population HNhps was recognized as the refugium during the Pleistocene ice ages, and populations in Guangdong were speculated to expand from Hunan^23^. In conjunction with the MAXENT predictions, the population genetic data will be used to develop an ex situ conservation action plan for *C. oliveri*.

In summary, this is the first study examining the adaptive loci, relationship between outliers and climatic factors, and the underlying mechanisms of local adaptation in *C. oliveri*. Our results indicated that *C. oliveris* exhibits remarkable adaptations to temperature and precipitation. Global warming may profoundly affect its viability and distribution. In the next steps we will dissect the adaptive value of the identified loci by sequencing and gene analysis in order to further understand the adaptation of *C. oliveri* to climatic factors.

## Methods

### Sample collection

Twenty-two naturally fragmented populations of *Cephalotaxus oliveri* were collected from the whole distribution range of the species (Table 1). Population samples contained five to fifteen individuals (Table 1). All selected populations except JXxs, JXyf, and GDdxs had 15 individuals, which were randomly sampled with 10–20 m interval. If the population size was less than 15, all individuals were collected. Young, healthy leaves were collected and dried in silica gel in zip-lock plastic bags until DNA extraction. Vouchers were deposited at the Herbarium of Sun Yat-sen University (SYSU), Guangzhou, China.

### Climatic data collection

Climatic layers at 2.5′ resolution for current conditions were obtained from the WorldClim database (http://www.worldclim.org/)^68^. The 19 bioclimatic variables were extracted by DIVA-GISv7.5 software (http://www.diva-gis.org/) in terms of informatic characterization of *C. oliveri*. The bioclimatic variables included annual mean temperature (Bio 1), mean diurnal range (Bio 2), Isothermality (Bio 3), temperature seasonality (Bio 4), max temperature of warmest month (Bio 5), min temperature of coldest month (Bio 6), temperature annual range (Bio 7), mean temperature of wettest quarter (Bio 8), mean temperature of driest quarter (Bio 9), mean temperature of warmest month (Bio 10), mean temperature of coldest month (Bio 11), annual precipitation (Bio 12), precipitation of wettest month (Bio 13), precipitation of driest month (Bio 14), precipitation seasonality (Bio 15), precipitation of wettest quarter (Bio 16), precipitation of driest quarter (Bio 17), precipitation of warmest quarter (Bio 18), and precipitation of coldest quarter (Bio 19) (Supplementary Table S1).

### DNA extraction

We extracted genomic DNA from tissues using a modified cetyltrimethyl ammonium bromide (CTAB) method with −20 °C propanone pretreatment to eliminate polysaccharides, which was successful for conifers^34^,^69^. The DNA was stored at −20 °C until further use.

### ISSR amplification

To cover the widest genomic region and ensure high-quality reproducible bands, an initial screening was developed using one individuals randomly obtained from HNhng population. Twenty-one primers were screened from 100 primers (UBC primer set #9) of the Biotechnology Laboratory, University of British Columbia. PCR amplification was carried out in a total volume of 20 μl consisting of 20 ng of template DNA, 10 mM Tris-HCl (pH 8.3) reaction buffer, 50 mM KCl, 2.0 mM MgCl_2_, 0.25 mM dNTPs, 0.24 μM primer, 1.5 units of *Taq* polymerase, and DNA-free water. In an ABI veriti thermocycler, PCR started with an initial denaturation at 94 °C for 5 min followed by 40 cycles with 94 °C for 30 s, 53 °C–54 °C for 45 s and 72 °C for 90 s, and ended with a final extension of 7 min at 72 °C. DNA quality and quantity were estimated using 1.7% agarose gel in TAE 1X buffer, stained with Ethidium Bromide. Additionally, 100 bp ladder and negative and positive controls were loaded and run at constant voltage (135 V) for 95 min. After running, the gels were UV visualized and recorded using a Gel Doc 2000 Camera.

### Data Analysis

Unambiguous ISSR fragments were transformed into 01 character matrix (1 = presence, 0 = absence).

POPGENE ver 1.31 was used to calculate genetic parameters. The estimates included the percentage of polymorphic loci (P), Nei’s (1973) gene diversity (H), Shannon’s information index (I), total gene diversity (Ht), and gene differentiation (G_*ST*_)^70^.

The variation among and within 22 populations were performed by analysis of molecular variance (AMOVA) with 1000 permutations using ARLEQUIN version 3.0^71^,^72^. Using the same software, a Mantel test^72^ was conducted to analyze the relationship between pairwise population genetic and geographic distances. Genetic distances were computed as pairwise *F*_ST_ values between all pairs of populations^73^. The unweighted pair group method (UPGMA) based on Nei’s unbiased (1978) genetic distance^74^ was performed using the TFPGA 1.3 program for constructing a dendrogram to reveal the genetic relationship among populations^75^. Bootstrap values for nodes were estimated based on 1000 replications.

To infer population structure, we assigned individuals and populations to clusters by using the model-based program STRUCTURE 2.3^76^. Thirty independent runs for each value of *K* = 1–27 were conducted to estimate the number of clusters (*K*) with maximum likelihood with the following settings: admixture model, correlated allele frequencies, burn-in length of 100 000, MCMC repetitions of 1000^77^, and ten times of iterations. A best-estimated *K* was defined both using log probabilities [Pr (X|K)] and hoc statistic Δ*K*^78^.

To identify loci under selection, software Dfdist and Bayescan were used to ensure truly adaptive regions of the genome. The software Dfdist was applied to simulate a null distribution of *F*_ST_ values under an island model, which was insensitive to population structure, demographic structure and mutation level. Simulations were computed with a mean *F*_ST_ similar to the trimmed mean *F*_ST_, which was calculated by excluding 30% of the most extreme *F*_ST_ values observed in the empirical dataset. We compared the distributions of the *F*_ST_ values over all loci to null hypothesis of neutral evolution. Loci with a high or low *F*_ST_ value were considered as potentially under selection. In this study, we simulated the neutral distribution of *F*_ST_ with 50 000 iterations at the 99.5% confidence level. Bayescan developed by Foll and Gaggiotti^27^ is an *F*_ST_ based model, which uses reversible jump MCMC and estimation of the Bayesian posterior probability^26^. It searches for loci with extreme *F*_ST_ values. Large *F*_ST_ is then interpreted as signature of local adaptation. We calculated outliers using a burn-in of 50 000 iterations, a thinning interval of 20, and a sample size of 5 000.

Linkage disequilibrium (LD) between all pairs of ISSR loci was calculated by the squared allele frequency correlation coefficient (*r*^2^) implemented in TASSEL 2.2 (Trait Analysis by aSSociation, Evolution, and Linkage)^79^. The pair-wise significance was computed by 1,000 permutations after removal of loci with rare alleles (f < 0.05).

The evaluated estimates included the standardized disequilibrium coefficient (*D*’), as well as the squared correlation coefficient (*r*^*2*^) and *p* values based on Fisher’s exact test. *D*’ determined whether recombination had occurred between a pair of alleles^80^. The critical value of *r*^2^ was the conventional 0.1^81^. Statistical tests for each *r*^2^ were provided by the *p* value calculated in TASSEL.

To investigate the association between ISSR genetic data and climatic variables, SAM was employed (available at http://www.econogene.eu/software/sam). The likelihood ratio (*G*) and Wald tests were used to determine the significance of the models. The null hypothesis was designed so that the above two statistical parameters conformed to a normal distribution. A model was considered significant only if the null hypothesis was rejected by both statistical tests at the 95 and 99.999% confidence level. The 310 ISSR markers in all 22 *C. oliveri* populations were examined against 19 climatic variables in the SAM analysis.

We used MAXENT 3.3.3k^82^ to predict distribution changes for *C. oliveri* as a result of climate warming. MAXENT is a program for maximum entropy modelling of the geographical distributions of species; it combines presence-only data with ecological-climatic layers to predict suitable areas. For current distribution, we downscaled climate grids for the periods 1950–2000. In addition to sample locations in this study, we also collected the distribution records of *C. oliveri* from the Chinese Virtual Herbarium (http://www.cvh.org.cn/). After removing duplicate records, it remained a total of 57 records of *C. oliveri* that were used to generate the distribution model by using 19 bioclimatic data layers from the WorldClim database (http://www.worldclim.org) at 2.5-arcmin resolution (Supplementary Table S1).

We selected the Hadley Global Environment Model 2 (HadGEM2-ES) as a general circulation model under two climate scenarios (IPCC-CMIP5 RCP 4.5/8.5) to ensure the accuracy of evaluation. The RCP 8.5 scenario represents a higher predicted greenhouse gas emission than RCP 4.5^83^ (Supplementary Tables S2–S3).

MAXENT was run according to the following settings: random test percentage = 25; regularization multiplier = 1; convergence threshold = 0.00001; maximum iterations = 1000 and averaged across 10 cross-validation runs. Ultimately, variable importance was determined by a jackknife test. We evaluated the accuracy of each model prediction by calculating the area under the curve (AUC) values. AUC is an efficient indicator of model performance^84^. AUC values > 0.9 indicates high reliability of the model^85^.

## Additional Information

**How to cite this article**: Wang, T. *et al*. Local adaptation to temperature and precipitation in naturally fragmented populations of *Cephalotaxus oliveri*, an endangered conifer endemic to China. *Sci. Rep.* **6**, 25031; doi: 10.1038/srep25031 (2016).

## Supplementary Material

Supplementary Information

## Figures and Tables

**Table 1:** Origin locations of sampled populations and parameters of genetic diversity revealed by ISSR in *Cephalotaxus oliveri*.

Province	Population	Population abbreviation	Geographical coordinate	Altitude (m)	Sample Size	Number of loci	Number of polymorphic loci	Percentage of polymorphic loci	Nei’s gene diversity	Shannon’s index
Chongqing	Jin Fo Shan	CQjfs	29°01′N 107°05′E	650	15	185	102	0.5514	0.2007	0.2973
	Liang Ping Zhu Shan	CQzs	30°39′N 107°32′E	530	15	193	133	0.6891	0.2156	0.3291
Yunnan	Da Wei Shan	YNdws	27°37′N 113°52′E	2109	15	185	118	0.6378	0.2249	0.334
Sichuan	E Mei Shan	SCems	29°33′N 103°23′E	980	15	173	97	0.5607	0.2089	0.3082
Jiangxi	An Fu	JXaf	27°13′N 114°11′E	420	15	180	131	0.7278	0.2477	0.3712
	Yi Feng	JXyf	28°37′N 114°54′E	741	8	155	62	0.4	0.1477	0.2186
	Xiu Shui	JXxs	28°46′N 114°46′E	307	5	141	26	0.1844	0.0798	0.1141
Hunan	De Hang	HNdh	28°21′N 109°35′E	429	15	190	101	0.5316	0.1782	0.2672
	Hu Ping Shan	HNhps	29°57′N 110°38′E	428–561	15	177	106	0.5989	0.2302	0.3373
	Hui Long	HNhl	28°54′N 110°10′E	399	15	199	136	0.6834	0.2346	0.3514
	Yong Mao	HNym	28°58′N 110°18′E	534	15	184	145	0.788	0.2769	0.4125
	Ha Ni Gong	HNhng	28°56′N 109°57′E	305	15	187	115	0.615	0.2098	0.3145
Guizhou	Wu Yang He	GZwyh	27°03′N 108°18′E	550	15	184	115	0.625	0.2235	0.3324
	Da Sha He	GZdsh	29°04′N 107°24′E	700	15	174	117	0.6724	0.2414	0.359
	Fan Jing Shan	GZfjs	27°49′N 108°36′E	860	15	166	80	0.4819	0.1643	0.246
Guangdong	Dan Xia Shan	GDdxs	25°03′N 113°45′E	800	8	150	67	0.4467	0.1792	0.2607
Hubei	Chang Yang	HBcy	30°43′N 110°54′E	420	15	185	117	0.6324	0.2203	0.3293
	Long Dong	HBld	34°40′N 111°02′E	342	15	176	91	0.517	0.1731	0.2615
	La Mei Xia	HBlmx	30°39′N 111°03′E	307	15	179	104	0.581	0.2067	0.3072
	Chai Bu Xi	HBcbx	30°11′N 111°01′E	248	15	187	113	0.6043	0.2076	0.3108
	Hou He	HBhh	30°05′N 110°40′E	440	15	174	86	0.4943	0.1772	0.2629
	Zi Gui Si Xi	Hbzg	30°43′N 111°54′E	248	15	182	120	0.6593	0.2141	0.3234
Total					306	269	251	0.9331	0.2214	0.3527

**Table 2:** AMOVA results for *Cephalotaxus oliveri* populations.

Source of variation	d.f.	Sum of squares	Variance components	Percentage of total variation	P-value	F statistics
Among regions	7	1657.683	2.84409	9.7	<0.001	*F*_CT_ = 0.09696
within regions	14	1972.364	8.76169	29.87	<0.001	*F*_SC_ = 0.33076
Within populations	284	5034.692	17.72779	60.44	<0.001	*F*_ST_ = 0.39565

**Table 3:** The results of linkage disequilibrium analyzed by TASSAL.

Locus	D’	*r*^*2*^	*p*
40 and 34	0.46113208	0.14939114	0.00000002
63 and 53	0.36157921	0.10927404	0.00000002
102 and 79	1.0000	0.10018378	0.00000035
161 and 143	0.78518778	0.12668338	0.00000102
165 and 130	0.82413793	0.2462112	0.00000117
165 and 135	0.50758618	0.24071099	0.00000004
165 and 144	0.91574889	0.13294548	0.00000001
165and 149	0.46627906	0.02834072	0.00837694
165 and 150	1.00	0.01317884	0.04848577
165 and 151	0.56534094	0.11084024	0.00000369
165 and 152	0.35944977	0.01535935	0.0493768
165 and 154	0.89827126	0.0709272	0.00000371
165 and 156	0.7589286	0.11122229	0.00000039
206 and 180	1.0000	0.16000	0.00000124
