# Supplementary material for: Local adaptation to temperature and precipitation in naturally fragmented populations of Cephalotaxus oliveri, an endangered conifer endemic to China
Source: Sci Rep. 2016 Apr 26;6:25031. doi: 10.1038/srep25031 (PMC4844950; doi:10.1038/srep25031)
Supplement: Supplementary Information [file srep25031-s1.pdf]

Local adaptation to temperature and precipitation in naturally fragmented populations of *Cephalotaxus oliveri* , an endangered conifer endemic to China

Ting Wang, Zhen Wang, Fan Xia & Yingjuan Su

Supplementary Table S1. Climate data of current distribution

Province	Population	Population abbreviation	Geographical coordinate	BIO1 (Annual Mean Temperature) (℃)	BIO2 (Mean Diurnal Range) (℃)
Chongqing	Jin Fo Shan	CQjfs	29°01′ N 107°05′ E	15.3	6.8
	Liang Ping Zhu Shan	CQzs	30°39′ N 107°32′ E	15.5	7.2
Yunnan	Da Wei Shan	YNdws	27°37′ N 113°52′ E	17.8	8.2
Sichuan	E Mei Shan	SCems	29°33′ N 103°23′ E	15.2	7.5
Jiangxi	An Fu	JXaf	27°13′ N 114°11′ E	16.1	7.9
	Yi Feng	JXyf	28°37′ N 114°54′ E	16.1	8.7
	Xiu Shui	JXxs	28°46′ N 114°46′ E	14.6	8.4
Hunan	De Hang	HNdh	28°21′ N 109°35′ E	15.1	8.1
	Hu Ping Shan	HNhps	29°57′ N 110°38′ E	12.8	7.4
	Hui Long	HNhl	28°54′ N 110°10′ E	15.9	8.2
	Yong Mao	HNym	28°58′ N 110°18′ E	14.3	7.8
	Ha Ni Gong	HNhng	28°56′ N 109°57′ E	15.8	8.2
Guizhou	Wu Yang He	GZwyh	27°03′ N 108°18′ E	16.6	8.6
	Da Sha He	GZdsh	29°04′ N 107°24′ E	14.1	6.8
	Fan Jing Shan	GZfjs	27°49′ N 108°36′ E	14.5	7.7
Guangdong	Dan Xia Shan	GDdxs	25°03′ N 113°45′ E	20.4	8.5
Hubei	Chang Yang	HBcy	30°43′ N 110°54′ E	13	7.9
	Long Dong	HBld	34°40′ N 111°02′ E	13.8	11.2
	La Mei Xia	HBlmx	30°39′ N 111°03′ E	16.5	8.4
	Chai Bu Xi	HBcbx	30°11′ N 111°01′ E	14.8	7.8
	Hou He	HBhh	30°05′ N 110°40′ E	14.4	7.7
	Zi Gui Si Xi	Hbzg	30°43′ N 111°54′ E	16.8	8.4

BIO3 (Isothermality)	BIO4 (Temperature Seasonality)	BIO5 (Max Temperature of Warmest Month) (℃)	BIO6 (Min Temperature of Coldest Month) (℃)	BIO7 (Temperature Annual Range) (℃)	BIO8 (Mean Temperature of Wettest Quarter) (℃)	BIO9 (Mean Temperature of Driest Quarter) (℃)
24	718.1	29.7	2.2	27.5	22.3	5.7
25	733.1	30.6	1.8	28.8	24.3	5.7
25	828.3	34.1	2	32.1	22	8.8
28	640.3	28	2	26	22.5	6.4
25	791.3	31.6	0.8	30.8	20.2	7.6
27	811.6	32.1	0.1	32	20	7.4
26	794.5	30.2	-1.2	31.4	18.5	6
27	778.5	30.2	0.3	29.9	22.7	4.6
25	759.9	27.3	-1.7	29	20.3	2.6
26	790.6	31.3	0.8	30.5	23.7	5.4
26	775.7	29.3	-0.5	29.8	21.9	3.9
27	784.1	31.1	0.8	30.3	23.4	5.3
29	748.3	31.8	2.3	29.5	23.9	6.5
24	716.6	28.3	0.9	27.4	21	4.4
26	751.9	29.4	0.4	29	22	4.4
29	708.9	34.2	5.6	28.6	24.1	12.7
26	775.5	27.9	-2	29.9	22.8	2.7
28	989.7	32.1	-6.7	38.8	24.5	0.4
27	805.4	31.9	0.9	31	26.6	5.7
26	788.2	29.8	-0.2	30	22.6	4.3
25	775.7	29.3	-0.4	29.7	22.1	4.1
26	837.3	32.4	0.6	31.8	27.3	5.7

BIO10 (Mean Temperature of Warmest Quarter) (℃)	BIO11 (Mean Temperature of Coldest Quarter) (℃)	BIO12 (Annual Precipitation) (mm)	BIO13 (Precipitation of Wettest Month) (mm)	BIO14 (Precipitation of Driest Month) (mm)	BIO15 (Precipitation Seasonality)	BIO16 (Precipitation of Wettest Quarter) (mm)
24.3	5.7	1170	179	21	59	511
24.7	5.7	1289	200	19	61	547
28	6.7	1569	230	49	45	659
23	6.4	1376	336	13	93	823
25.9	5.5	1646	241	51	46	699
26.2	5.3	1446	231	43	51	642
24.5	4	1600	254	45	50	702
24.8	4.6	1380	213	34	53	581
22.3	2.6	1430	211	32	54	606
25.8	5.4	1451	246	36	55	640
24	3.9	1502	241	37	54	650
25.6	5.3	1446	242	34	56	637
25.8	6.5	1156	198	27	56	506
23	4.4	1237	182	23	58	531
23.9	4.4	1239	198	28	56	523
28.9	10.9	1520	252	43	57	707
22.8	2.7	1220	205	25	57	524
26.3	0.4	487	102	7	77	261
26.6	5.7	1135	206	22	60	517
24.7	4.3	1290	204	28	55	548
24.2	4.1	1339	206	29	55	572
27.3	5.7	1053	180	24	57	470

BIO17 (Precipitation of Driest Quarter) (mm)	BIO18 (Precipitation of Warmest Quarter) (mm)	BIO19 (Precipitation of Coldest Quarter) (mm)
69	484	69
65	525	65
193	483	221
47	796	47
194	515	227
163	485	184
175	549	197
112	534	112
110	587	110
120	586	120
125	597	125
113	589	113
87	462	87
74	500	74
87	483	87
139	517	190
91	524	91
21	242	21
81	517	81
101	548	101
103	561	103
85	470	85

Supplementary Table S2. Climate data of Scenario RCP4.5

Province	Population	Population abbreviation	Geographical coordinate	BIO1 (Annual Mean Temperature) (℃)	BIO2 (Mean Diurnal Range) (℃)
Chongqing	Jin Fo Shan	CQjfs	29°01′ N 107°05′ E	18.2	7.2
	Liang Ping Zhu Shan	CQzs	30°39′ N 107°32′ E	18.3	7.5
Yunnan	Da Wei Shan	YNdws	27°37′ N 113°52′ E	21	8.6
Sichuan	E Mei Shan	SCems	29°33′ N 103°23′ E	18.5	7.8
Jiangxi	An Fu	JXaf	27°13′ N 114°11′ E	19.3	8.3
	Yi Feng	JXyf	28°37′ N 114°54′ E	19.4	9.1
	Xiu Shui	JXxs	28°46′ N 114°46′ E	17.9	8.9
Hunan	De Hang	HNdh	28°21′ N 109°35′ E	18.1	8.3
	Hu Ping Shan	HNhps	29°57′ N 110°38′ E	15.7	7.5
	Hui Long	HNhl	28°54′ N 110°10′ E	19	8.4
	Yong Mao	HNym	28°58′ N 110°18′ E	17.4	8
	Ha Ni Gong	HNhng	28°56′ N 109°57′ E	18.8	8.4
Guizhou	Wu Yang He	GZwyh	27°03′ N 108°18′ E	19.6	8.8
	Da Sha He	GZdsh	29°04′ N 107°24′ E	17	7.2
	Fan Jing Shan	GZfjs	27°49′ N 108°36′ E	17.5	8
Guangdong	Dan Xia Shan	GDdxs	25°03′ N 113°45′ E	23.3	8.6
Hubei	Chang Yang	HBcy	30°43′ N 110°54′ E	16	8
	Long Dong	HBld	34°40′ N 111°02′ E	16.8	11.3
	La Mei Xia	HBlmx	30°39′ N 111°03′ E	19.5	8.5
	Chai Bu Xi	HBcbx	30°11′ N 111°01′ E	17.8	8
	Hou He	HBhh	30°05′ N 110°40′ E	17.4	7.8
	Zi Gui Si Xi	Hbzg	30°43′ N 111°54′ E	19.8	8.7

BIO3 (Isothermality)	BIO4 (Temperature Seasonality)	BIO5 (Max Temperature of Warmest Month) (℃)	BIO6 (Min Temperature of Coldest Month) (℃)	BIO7 (Temperature Annual Range) (℃)	BIO8 (Mean Temperature of Wettest Quarter) (℃)	BIO9 (Mean Temperature of Driest Quarter) (℃)
25	741.8	32.9	4.9	28	25.4	8.3
25	763.5	34	4.5	29.5	25.6	8.2
26	836.2	37.5	5.6	31.9	25	16.6
30	650.7	31.5	5.7	25.8	26.1	9.6
27	795.3	34.9	4.3	30.5	23.2	15.1
28	825.5	35.8	3.7	32.1	23.1	15.1
28	810.6	33.8	2.5	31.3	21.5	13.6
27	787	33.3	3.5	29.8	25.7	7.5
25	775.2	30.6	1.4	29.2	23.2	5.5
27	801.1	34.6	4	30.6	26.6	8.3
26	785.5	32.5	2.7	29.8	24.9	6.9
27	796.1	34.4	3.9	30.6	26.4	8.2
30	752.7	34.4	5.4	29	26.9	9.4
25	739.3	31.4	3.6	27.9	24.1	7
27	759.7	32.1	3.5	28.6	24.9	7.3
31	691.6	36.8	9.1	27.7	26.8	19.9
26	791.9	31.1	0.9	30.2	23.7	5.6
29	1010.4	35.7	-3.3	39	28	3.4
27	822.1	35.2	3.8	31.4	29.7	8.6
26	802.8	33.1	2.8	30.3	25.6	7.2
26	790.9	32.7	2.7	30	25	6.9
27	853.2	35.7	3.5	32.2	30.5	8.6

BIO10 (Mean Temperature of Warmest Quarter) (℃)	BIO11 (Mean Temperature of Coldest Quarter) (℃)	BIO12 (Annual Precipitation) (mm)	BIO13 (Precipitation of Wettest Month) (mm)	BIO14 (Precipitation of Driest Month) (mm)	BIO15 (Precipitation Seasonality)	BIO16 (Precipitation of Wettest Quarter) (mm)
27.4	8.3	1336	225	23	66	632
27.9	8.2	1436	259	22	65	650
31.4	10	1737	260	60	48	739
26.3	9.6	1495	378	15	93	895
29.1	8.8	1814	270	62	49	774
29.7	8.6	1593	267	54	53	722
28	7.3	1761	290	58	52	789
27.8	7.5	1625	275	40	58	723
25.4	5.5	1737	278	39	59	783
28.9	8.3	1764	307	43	61	821
27.1	6.9	1831	301	45	59	837
28.6	8.2	1744	303	41	61	818
28.8	9.4	1324	250	30	60	597
26.1	7	1415	230	25	64	662
26.9	7.3	1429	249	33	60	630
31.6	14	1639	288	37	56	729
25.9	5.6	1422	249	28	58	620
29.7	3.4	536	136	8	81	290
29.7	8.6	1340	255	26	63	622
27.8	7.2	1559	264	34	59	700
27.2	6.9	1622	270	35	59	737
30.5	8.6	1281	233	28	62	588

BIO17 (Precipitation of Driest Quarter) (mm)	BIO18 (Precipitation of Warmest Quarter) (mm)	BIO19 (Precipitation of Coldest Quarter) (mm)
74	599	74
71	614	71
205	528	278
55	866	55
203	567	284
175	519	236
194	584	252
135	649	135
134	749	134
147	748	147
153	766	153
135	747	135
101	548	101
80	618	80
101	568	101
142	600	230
109	618	109
28	283	28
99	622	99
123	690	123
125	712	125
103	588	103

Supplementary Table S3. Climate data of Scenario RCP8.5

Province	Population	Population abbreviation	Geographical coordinate	BIO1 (Annual Mean Temperature) (℃)	BIO2 (Mean Diurnal Range) (℃)
Chongqing	Jin Fo Shan	CQjfs	29°01′ N 107°05′ E	19.9	7.3
	Liang Ping Zhu Shan	CQzs	30°39′ N 107°32′ E	20	7.5
Yunnan	Da Wei Shan	YNdws	27°37′ N 113°52′ E	23	8.8
Sichuan	E Mei Shan	SCems	29°33′ N 103°23′ E	20.2	7.9
Jiangxi	An Fu	JXaf	27°13′ N 114°11′ E	21.3	8.4
	Yi Feng	JXyf	28°37′ N 114°54′ E	21.4	9.3
	Xiu Shui	JXxs	28°46′ N 114°46′ E	19.9	9.1
Hunan	De Hang	HNdh	28°21′ N 109°35′ E	19.9	8.4
	Hu Ping Shan	HNhps	29°57′ N 110°38′ E	17.6	7.6
	Hui Long	HNhl	28°54′ N 110°10′ E	20.8	8.4
	Yong Mao	HNym	28°58′ N 110°18′ E	19.2	8
	Ha Ni Gong	HNhng	28°56′ N 109°57′ E	20.6	8.4
Guizhou	Wu Yang He	GZwyh	27°03′ N 108°18′ E	21.3	8.8
	Da Sha He	GZdsh	29°04′ N 107°24′ E	18.6	7.3
	Fan Jing Shan	GZfjs	27°49′ N 108°36′ E	19.2	8
Guangdong	Dan Xia Shan	GDdxs	25°03′ N 113°45′ E	25.1	8.4
Hubei	Chang Yang	HBcy	30°43′ N 110°54′ E	17.8	8
	Long Dong	HBld	34°40′ N 111°02′ E	18.5	11
	La Mei Xia	HBlmx	30°39′ N 111°03′ E	21.3	8.5
	Chai Bu Xi	HBcbx	30°11′ N 111°01′ E	19.6	8
	Hou He	HBhh	30°05′ N 110°40′ E	19.2	7.9
	Zi Gui Si Xi	Hbzg	30°43′ N 111°54′ E	21.6	8.7

BIO3 (Isothermality)	BIO4 (Temperature Seasonality)	BIO5 (Max Temperature of Warmest Month) (℃)	BIO6 (Min Temperature of Coldest Month) (℃)	BIO7 (Temperature Annual Range) (℃)	BIO8 (Mean Temperature of Wettest Quarter) (℃)	BIO9 (Mean Temperature of Driest Quarter) (℃)
25	761.1	34.8	6.5	28.3	27.2	9.8
25	786.2	36	6.1	29.9	27.5	9.7
27	857.3	40	7.5	32.6	26.8	18.6
30	649.9	33.1	7.5	25.6	27.8	11.4
27	814.3	37.3	6.2	31	24.9	17.1
28	849.9	38.8	5.7	33.1	24.8	17
28	834.8	36.8	4.4	32.4	23.3	15.6
27	807.1	35.6	5.1	30.5	23.7	9.3
25	802.5	32.9	3	29.9	25.2	9.3
26	824.7	37	5.7	31.3	28.5	12.3
26	809.4	34.9	4.4	30.5	26.8	10.8
26	819.7	36.8	5.6	31.3	28.3	9.9
30	770.8	36.4	7.1	29.3	28.7	11.1
25	759	33.4	5.2	28.3	25.9	8.6
27	779.6	34.2	5.1	29.1	26.7	9
30	700.5	38.6	11	27.6	28.6	21.7
25	817	33.3	2.5	30.8	25.6	9.3
28	1018.3	37.5	-1.3	38.8	29.9	5.1
26	847.7	37.5	5.5	32	29.4	12.4
25	827.2	35.4	4.5	30.9	27.5	11
25	818.1	35	4.3	30.7	26.9	10.7
26	874.8	38	5.2	32.8	30.1	12.4

BIO10 (Mean Temperature of Warmest Quarter) (℃)	BIO11 (Mean Temperature of Coldest Quarter) (℃)	BIO12 (Annual Precipitation) (mm)	BIO13 (Precipitation of Wettest Month) (mm)	BIO14 (Precipitation of Driest Month) (mm)	BIO15 (Precipitation Seasonality)	BIO16 (Precipitation of Wettest Quarter) (mm)
29.3	9.8	1349	237	21	69	651
29.9	9.7	1453	246	21	66	658
33.8	12	1750	269	54	47	748
28	11.4	1545	380	16	95	945
31.5	10.8	1821	277	55	47	775
32.2	10.6	1617	251	47	51	721
30.5	9.4	1789	277	50	50	790
29.9	9.3	1677	302	35	62	767
27.6	7.2	1779	304	36	60	806
31	10.1	1791	307	39	62	841
29.2	8.7	1857	318	40	61	856
30.8	9.9	1776	307	37	63	841
30.8	11.1	1394	262	29	64	645
28	8.6	1428	244	23	68	681
28.9	9	1496	278	29	65	681
33.5	15.9	1651	288	37	55	718
28.1	7.3	1502	244	28	60	664
31.5	5.1	584	136	10	76	303
31.9	10.3	1409	247	25	63	639
30	8.9	1613	266	32	60	726
29.4	8.6	1665	278	33	60	759
32.7	10.3	1352	222	28	63	591

BIO17 (Precipitation of Driest Quarter) (mm)	BIO18 (Precipitation of Warmest Quarter) (mm)	BIO19 (Precipitation of Coldest Quarter) (mm)
68	619	68
69	623	69
203	385	288
57	907	57
205	427	291
171	352	247
189	408	266
133	664	133
136	718	140
145	733	149
150	746	155
136	737	136
99	592	99
74	636	74
97	605	97
137	462	227
108	625	114
35	289	35
100	624	104
123	669	130
127	684	131
104	587	110
